# Association of myopia and astigmatism with postoperative ocular high order aberration after small incision lenticule extraction

**DOI:** 10.1186/s12886-024-03475-w

**Published:** 2024-05-13

**Authors:** Yifan Du, Yu Di, Shan Yang, Fei Mo, Ge Cui, Di Chen, Ying Li

**Affiliations:** Department of Ophthalmology, Peking Union Medical College Hospital, Chinese Academy of Medical Sciences & Peking Union Medical College, Beijing, People’s Republic of China

**Keywords:** Higher-order aberrations, Myopia, Astigmatism, Small incision lenticule extraction

## Abstract

**Objective:**

To investigate the correlation between higher-order aberrations (HOA) after small incision lenticule extraction (SMILE) and the severity of myopia and astigmatism, along with the relevant factors. These findings will provide valuable insights for decreasing the occurrence of HOA after SMILE and enhancing visual quality.

**Methods:**

A total of 75 patients (150 eyes) with myopia and astigmatism who underwent SMILE were categorized into four groups based on the severity of myopia and astigmatism: Myopia Group 1 (Group M1, spherical diopter ranged from -1.00 D to -4.00 D), Myopia Group 2 (Group M2, spherical diopter ranged from -4.10 D to -10.00 D), Astigmatism Group 1 (Group A1, cylindrical diopter ranged from 0 D to -1.00 D), and Astigmatism Group 2 (Group A2, cylindrical diopter ranged from -1.10 D to -3.00 D). A comprehensive assessment was performed to examine the association between HOA and various relevant factors, including a detailed analysis of the subgroups.

**Results:**

Group M1 had significantly lower levels of total eye coma aberration (CA), corneal total HOA (tHOA), internal tHOA, and vertical CA (${{\text{Z}}}_{3}^{-1}$) after SMILE than Group M2 (*P* < 0.05). Similarly, Group A1 had significantly lower levels of total eye tHOA, CA, trefoil aberration (TA), corneal tHOA, TA, and vertical TA (${{\text{Z}}}_{3}^{-3}$) after SMILE than Group A2 (*P* < 0.05). Pearson correlation analysis indicated a statistically significant positive relationship between the severity of myopia/astigmatism and most HOA (*P* < 0.05). Subgroup evaluations demonstrated a notable increase in postoperative HOA associated with myopia and astigmatism in Groups M2 and A2 compared with the control group. Lenticule thickness, postoperative central corneal thickness (CCT), postoperative uncorrected distance visual acuity (UDVA), and postoperative corneal Km and Cyl were strongly correlated with most HOA. Age, eyes, and postoperative intraocular pressure (IOP) were only associated with specific HOA.

**Conclusion:**

HOA positively correlated with the severity of myopia and astigmatism after SMILE. However, this relationship was not linear. HOA after SMILE was influenced by various factors, and additional specialized investigations are required to establish its clinical importance.

**Supplementary Information:**

The online version contains supplementary material available at 10.1186/s12886-024-03475-w.

## Introduction

Scientific findings have reported a significant increase in the prevalence of myopia worldwide over the past few years [1]. It is estimated that by 2050, more than 4.76 billion people globally will have myopia, and roughly 940 million of them will have myopia of -5.00 diopter (D) or greater [2]. The prevalence of this condition has resulted in significant anguish in patients’ everyday experiences. Therefore, corneal refractive surgery has emerged as an effective solution to this problem [3, 4]. Small incision lenticule extraction (SMILE) is a modern technique for refractive surgery that was first described by Sekundo et al. in 2008 [5]. Following this, many studies have reported an outstanding improvement in visual acuity after SMILE. It offers the advantages of a convenient surgical procedure, stable postoperative recovery, and excellent treatment results. However, a significant proportion of patients still expressed dissatisfaction with their visual quality after undergoing SMILE. The post-SMILE patient group experienced an increasing number of visual problems, which became an important factor in evaluating the effectiveness of modern refractive surgery [6].

The increasing advancement and popularity of visual analysis technologies have highlighted higher-order aberrations (HOA) as the main factor affecting vision quality, particularly night vision symptoms [7]. This pertains to a type of optical distortion that occurs in the third order or above and cannot be corrected by wearing glass. It includes different types of aberration, such as spherical aberration (SA), coma aberration (CA), and trefoil aberration (TA). Studies have suggested that this anomaly may be linked to factors such as age, intraocular pressure (IOP), race, or refractive error [8–11]. Research on refractive surgery has consistently shown that corneal refractive surgery, including SMILE, can induce HOA. In a study conducted by Sekundo et al., an increase in total HOA (tHOA) was observed after SMILE [12]. However, Kwak et al. noted that SMILE does not have a substantial impact on SA [13]. Therefore, no definitive result has been reached regarding the impact of myopia and astigmatism after SMILE on HOA [12–15]. This study aimed to examine the relationship between the severity of myopia/astigmatism and the development of postoperative ocular HOA after SMILE, and to identify potentially relevant factors. Ultimately, the goal was to provide recommendations for reducing the induction of postoperative HOA and enhancing the visual quality after SMILE.

## Methods

### Subjects

This retrospective study included a cohort of patients who underwent SMILE at Peking Union Medical College Hospital between October 2020 and October 2023. The patients underwent comprehensive preoperative eye examination and were regularly reexamined after surgery. The inclusion criteria for this study were: 1) the ages of these participants ranged from 18 to 40 years old; 2) the preoperative corrected distance visual acuity (CDVA) should be 20/25 or better; 3) subjects with the spherical diopter (DS) of subjective refraction less than -10.00 D and cylindrical diopter (DC) less than -3.00 D, the refractive status has remained stable for the past two years; 4) subjects were not currently using topical punctual ophthalmic medications, have not undergone any ocular surgery in the past, had no history of any other ocular disease, and there were no contraindications to surgery identified during the outpatient evaluation; 5) there were no systemic disease or drug allergy that may adversely affect the eye and vision; 6) range of preoperative IOP measured by non-contact tonometer: 10–21 mmHg; 7) no active inflammation. Next, cases were classified into two different categories based on the severity of myopia and astigmatism, each of which consisted of two groups: Myopia Group 1 (Group M1, spherical diopter ranged from -1.00 D to -4.00 D) and Myopia Group 2 (Group M2, spherical diopter ranged from -4.10 D to -10.00 D); Astigmatism Group 1 (Group A1, cylindrical diopter ranged from 0 D to -1.00 D) and Astigmatism Group 2 (Group A2, cylindrical diopter ranged from -1.10 D to -3.00 D). This study strictly adhered to the principles of the Declaration of Helsinki and was formally approved by the Institutional Review Board of the Peking Union Medical College Hospital. Informed consent was obtained from all participants.

### Data collection

All selected patients underwent an exhaustive ophthalmological examination before surgery, including CDVA, evaluation of the dominant eye, automatic refraction, cycloplegic refraction, slit-lamp biomicroscopy, non-contact intraocular tonometry (CT-1P; Topcon, Japan), dilated pupil fundus examination, optic disc stereography (VX-10; Kowa Optimed, Tokyo, Japan), central corneal thickness (CCT) measurement (Orbscan 73 II; Bausch&Lomb Surgical, Rochester, NY, USA), corneal topography (Pentacam HR; Oculus, Wetzlar, Germany), and optical coherence tomography (OCT, Spectralis SD-OCT; Heidelberg Engineering, Inc., Heidelberg, Germany). Each examination was performed by skilled ophthalmic technicians and carefully reviewed and integrated by professional ophthalmologists (Y.F.D. and S.Y.).

### Surgical procedure

The surgery was performed using the SMILE 3.0 procedure of the VisuMax femtosecond laser system (Carl Zeiss Meditec AG, Jena, Germany) with a repetition rate of 500 kHz and pulse energy of 110–120 nJ. The corneal cap was set at a thickness of 110–120 μm and diameter of 7.6 mm. The lenticule optical zone was 6.5 mm with 0.1 mm transition zone for astigmatism. The patient's head was adjusted to maintain a horizontal position and to correct the astigmatism axis. The patient's eye was positioned under the cone, and the patient was instructed to fixate on a blinking green light. Once in position, corneal suction ports are activated to fix the eye in this position. In this way, the patient aligns the visual axis and, hence, the corneal vertex to the vertex of the contact glass, which is centered on the laser system. A 3.0 mm incision was made at the 120-degree position from which the lenticule was extracted. The target refraction was the emmetropia. All surgeries were performed by the same experienced surgeon (Y.L.) and then by a specialized ophthalmologist (Y.D.) who collected relevant surgical information on these cases at the end of the procedure. Antimicrobial and anti-inflammatory treatments were routinely administered postoperatively, and examinations during the follow-up period including visual acuity, IOP, corneal topography, CDVA, and slit-lamp biomicroscopy were performed regularly.

### Measurement of ocular HOA

HOA and corneal topographic data were obtained using the iTrace wavefront analyzer and corneal topographer, both contained within one apparatus (iTrace, Tracey Technology, Houston, TX, USA). The HOA of all patients in this study was measured 3 months after SMILE. The participants were instructed to perform a blinking action to minimize interference from the tear film before measurement. Subsequently, they were focused and photographed for a period of 3 s, and the mean value was selected from the results of three high-quality experiments. The Schwiegerling formula was used to avoid the influence of different patients and pupil sizes on the measurements [16]. To ensure consistency of results as much as possible, the aberration measurements of all participants were subsequently converted to root mean square (RMS) values of the Zernike coefficient for the same pupil diameter (4 mm) using MATLAB (software version 7.6.0; The Math Works, Inc., Natick, MA) to allow for comparison. Finally, various data related to HOA were collated, including total HOA (tHOA), SA, CA, and TA from the total eye, cornea, and internal, as well as information for the total eye HOA obtained from ${{\text{Z}}}_{3}^{-3}$, ${{\text{Z}}}_{3}^{-1}$, ${{\text{Z}}}_{3}^{1}$, ${{\text{Z}}}_{3}^{3}$, ${{\text{Z}}}_{4}^{-4}$, ${{\text{Z}}}_{4}^{-2}$, ${{\text{Z}}}_{4}^{2}$, and ${{\text{Z}}}_{4}^{4}$.

### Statistical analysis

The SPSS statistical package (version 25.0; IBM SPSS Inc., Chicago, Illinois, USA) was used to evaluate the data. Continuous variables are presented as mean ± standard deviation, whereas discontinuous variables are presented as percentages (%). Using the Kolmogorov–Smirnov test, all data conformed to a normal distribution pattern. Differences in HOA among all groups were subsequently compared using independent sample t-tests, and the Bonferroni correction for multiple testing was used to reduce the rate of type I error. Pearson's correlation analysis was used to assess the relationship between myopia/astigmatism (DS and DC of subjective refraction) and individual HOA. Finally, the correlation between tHOA and these parameters was evaluated. Statistical significance was set at *P* < 0.05.

## Results

### Clinical characteristics of the subjects

A total of 75 subjects (150 eyes) were enrolled in this study, including 20 men (40 eyes) and 55 women (110 eyes). The mean age of the subjects was 28.79 ± 5.67 years. Both eyes of all patients in this cohort underwent SMILE, and their mean preoperative CCT was 536.34 ± 27.81 mm, mean preoperative IOP was 15.55 ± 2.92 mmHg, mean preoperative DS was -4.59 ± 1.38 D, mean preoperative DC was -0.83 ± 0.66 D, mean preoperative CDVA (logMAR) was -0.04 ± 0.04, mean preoperative Km was 43.60 ± 1.32 D, and the mean preoperative Cyl was 1.26 ± 0.71 D. Table 1 summarizes the main clinical characteristics of the cases included in this study.

**Table 1:** Clinical characteristics of study participants

Characteristics	Total	Group M1	Group M2	*P*	Group A1	Group A2	*P*
Number of eyes (n)	150	65	85		103	47	
Gender (male/female)	20/55	8/25	12/30	0.622	11/40	9/15	**0.030**
Eye (OD/OS)	75/75	28/37	47/38	0.140	51/52	24/23	0.861
Age (yrs)	28.79 ± 5.67	28.57 ± 4.89	28.95 ± 6.22	0.683	29.33 ± 5.65	27.60 ± 5.59	0.082
Preop CCT (μm)	536.34 ± 27.81	537.54 ± 29.71	535.42 ± 26.40	0.646	532.91 ± 24.96	539.85 ± 32.23	0.062
Preop IOP (mmHg)	15.55 ± 2.92	16.13 ± 2.92	14.94 ± 2.81	0.069	15.32 ± 3.11	16.06 ± 2.41	0.297
Preop sphere (D)	-4.59 ± 1.38	-3.35 ± 0.62	-5.53 ± 0.99	** < 0.001**	-4.50 ± 1.24	-4.78 ± 1.64	0.251
Preop cylinder (D)	-0.83 ± 0.66	-0.76 ± 0.60	-0.89 ± 0.71	0.249	-0.47 ± 0.35	-1.62 ± 0.49	** < 0.001**
Preop CDVA (logMAR)	-0.04 ± 0.04	-0.04 ± 0.04	-0.03 ± 0.04	0.751	-0.04 ± 0.04	-0.03 ± 0.04	0.194
Preop Km (D)	43.60 ± 1.32	43.58 ± 1.27	43.61 ± 1.37	0.910	43.51 ± 1.23	43.76 ± 1.47	0.371
Preop Cyl (D)	1.26 ± 0.71	1.20 ± 0.68	1.31 ± 0.73	0.429	0.90 ± 0.42	1.92 ± 0.64	** < 0.001**

*CDVA* Corrected distance visual acuity, *CCT* Central corneal thickness, *Cyl* Cylindrical Lens, *IOP* Intraocular pressure, *Km* Mean keratometry, *Preop* Preoperative. All values are displayed in the form of mean ± standard deviation. *P* values < 0.05 at t-test are presented in boldface

### Comparison of myopia and astigmatism groupings

**Fig. 1:**
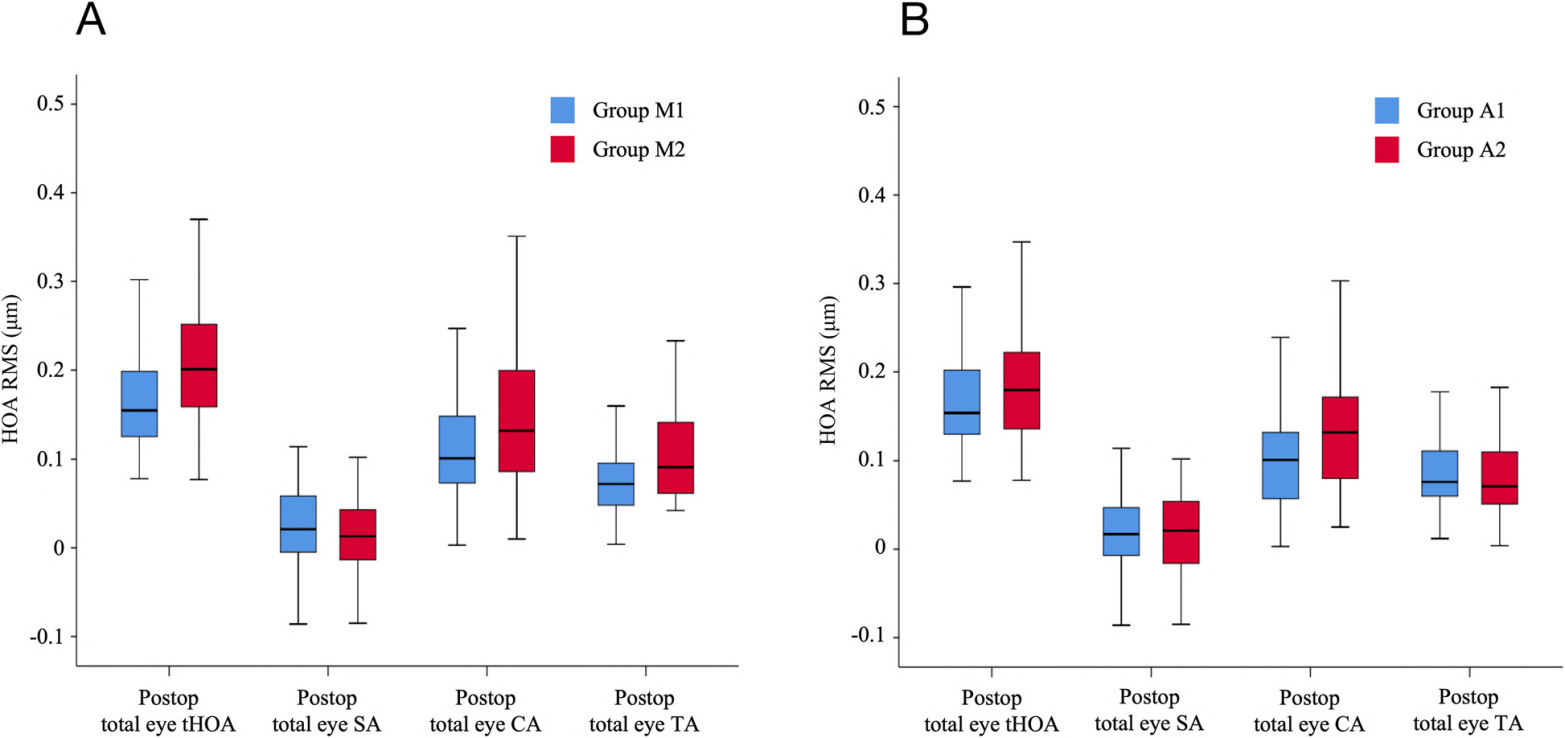
Comparison of post-op HOA between different groups. **A **Comparison of Group M1 (spherical diopter ranged from -1.0 D to -4.00 D) and Group M2 (spherical diopter ranged from -4.10 D to -10.00 D); **B **Comparison of Group A1 (cylindrical diopter ranged from 0 D to -1.00 D) and Group A2 (cylindrical diopter ranged from -1.10 D to -3.00 D)

In the comparison of myopia groups, Group M1 had a significantly lower (*P* < 0.05) total eye CA (0.112 ± 0.076 vs. 0.139 ± 0.077), corneal tHOA (0.192 ± 0.082 vs. 0.252 ± 0.135), corneal CA (0.133 ± 0.084 vs. 0.176 ± 0.110), internal tHOA (0.182 ± 0.086 vs. 0.219 ± 0.111), and ${{\text{Z}}}_{3}^{-1}$(vertical CA, -0.054 ± 0.104 vs. -0.093 ± 0.109) after SMILE than Group M2. Regarding the comparison of astigmatism groups, Group A1 compared with Group A2 had a significantly lower total eye tHOA (0.173 ± 0.073 vs. 0.214 ± 0.087), total eye CA (0.118 ± 0.071 vs. 0.148 ± 0.088), total eye TA (0.076 ± 0.040 vs. 0.104 ± 0.056), corneal tHOA (0.211 ± 0.104 vs. 0.260 ± 0.141), corneal TA (0.089 ± 0.066 vs. 0.133 ± 0.123), and ${{\text{Z}}}_{3}^{-3}$ (vertical TA, 0.015 ± 0.059 vs. 0.046 ± 0.085) after SMILE. Comparisons of the specific myopia and astigmatism groups are shown in Table 2 and Fig. 1.

**Table 2:** Comparison of HOA under different myopia and astigmatism groups

Parameters	Group M1	Group M2	t	*P*	Group A1	Group A2	t	*P*
Total eye HOA RMS								
tHOA	0.176 ± 0.082	0.193 ± 0.772	-1.357	0.177	0.173 ± 0.073	0.214 ± 0.087	-3.004	**0.003**
SA	0.024 ± 0.060	0.022 ± 0.053	0.266	0.791	0.025 ± 0.056	0.019 ± 0.056	0.650	0.517
CA	0.112 ± 0.076	0.139 ± 0.077	-2.061	**0.041**	0.118 ± 0.071	0.148 ± 0.088	-2.243	**0.026**
TA	0.086 ± 0.043	0.083 ± 0.050	0.392	0.695	0.076 ± 0.040	0.104 ± 0.056	-3.490	**0.001**
Cornea HOA RMS								
tHOA	0.192 ± 0.082	0.252 ± 0.135	-3.178	**0.002**	0.211 ± 0.104	0.260 ± 0.141	-2.388	**0.018**
SA	0.033 ± 0.058	0.013 ± 0.075	1.798	0.074	0.019 ± 0.072	0.027 ± 0.060	-0.642	0.522
CA	0.133 ± 0.084	0.176 ± 0.110	-2.609	**0.010**	0.151 ± 0.100	0.170 ± 0.105	-1.090	0.277
TA	0.087 ± 0.054	0.114 ± 0.109	-1.837	0.068	0.089 ± 0.066	0.133 ± 0.123	-2.825	**0.005**
Internal HOA RMS								
tHOA	0.182 ± 0.086	0.219 ± 0.111	-2.200	**0.029**	0.194 ± 0.098	0.223 ± 0.108	-1.630	0.105
SA	-0.009 ± 0.076	0.009 ± 0.084	-1.337	0.183	0.006 ± 0.081	-0.008 ± 0.079	1.008	0.315
CA	0.104 ± 0.080	0.122 ± 0.088	-1.298	0.196	0.109 ± 0.089	0.126 ± 0.075	-1.122	0.264
TA	0.087 ± 0.045	0.103 ± 0.084	-1.340	0.182	0.093 ± 0.057	0.102 ± 0.093	-0.667	0.505
Other total eye HOA RMS								
${{\text{Z}}}_{3}^{-3}$	0.023 ± 0.070	0.026 ± 0.070	-0.286	0.776	0.015 ± 0.059	0.046 ± 0.085	-2.601	**0.010**
${{\text{Z}}}_{3}^{-1}$	-0.054 ± 0.104	-0.093 ± 0.109	2.227	**0.027**	-0.073 ± 0.098	-0.082 ± 0.129	0.503	0.616
${{\text{Z}}}_{3}^{1}$	-0.006 ± 0.070	-0.004 ± 0.070	-0.140	0.889	-0.006 ± 0.064	-0.002 ± 0.082	-0.306	0.760
${{\text{Z}}}_{3}^{3}$	-0.002 ± 0.063	0.011 ± 0.062	-1.323	0.188	0.000 ± 0.061	0.017 ± 0.066	-1.584	0.115
${{\text{Z}}}_{4}^{-4}$	0.003 ± 0.028	0.000 ± 0.027	0.264	0.792	0.002 ± 0.024	-0.000 ± 0.034	0.420	0.675
${{\text{Z}}}_{4}^{-2}$	0.000 ± 0.019	0.005 ± 0.021	-1.441	0.152	0.002 ± 0.020	0.005 ± 0.023	-0.744	0.458
${{\text{Z}}}_{4}^{2}$	0.003 ± 0.035	0.009 ± 0.031	-0.952	0.343	0.009 ± 0.033	0.001 ± 0.033	1.335	0.184
${{\text{Z}}}_{4}^{4}$	0.018 ± 0.026	0.019 ± 0.026	-0.210	0.834	0.021 ± 0.024	0.014 ± 0.029	1.558	0.121

*CA* Coma aberration, *HOA* High order aberration, *RMS* Root mean square, *SA* Spherical aberration, *TA* Trefoil aberration, *tHOA* Total high order aberration; ${{\text{Z}}}_{3}^{-3}$ = vertical trifoil aberration; ${{\text{Z}}}_{3}^{-1}$ = vertical coma aberration; ${{\text{Z}}}_{3}^{1}$ = horizontal coma aberration; ${{\text{Z}}}_{3}^{3}$ = oblique trifoil aberration; ${{\text{Z}}}_{4}^{-4}$ = oblique quadrafoil aberration; ${{\text{Z}}}_{4}^{-2}$ = oblique secondary astigmatism; ${{\text{Z}}}_{4}^{2}$ = vertical secondary astigmatism; ${{\text{Z}}}_{4}^{4}$ = vertical quadrafoil aberration. All values are displayed in the form of mean ± standard deviation. *P* values < 0.05 at t-test are presented in boldface

### Relationship between the degree of myopia/Astigmatism and HOA

**Fig. 2:**
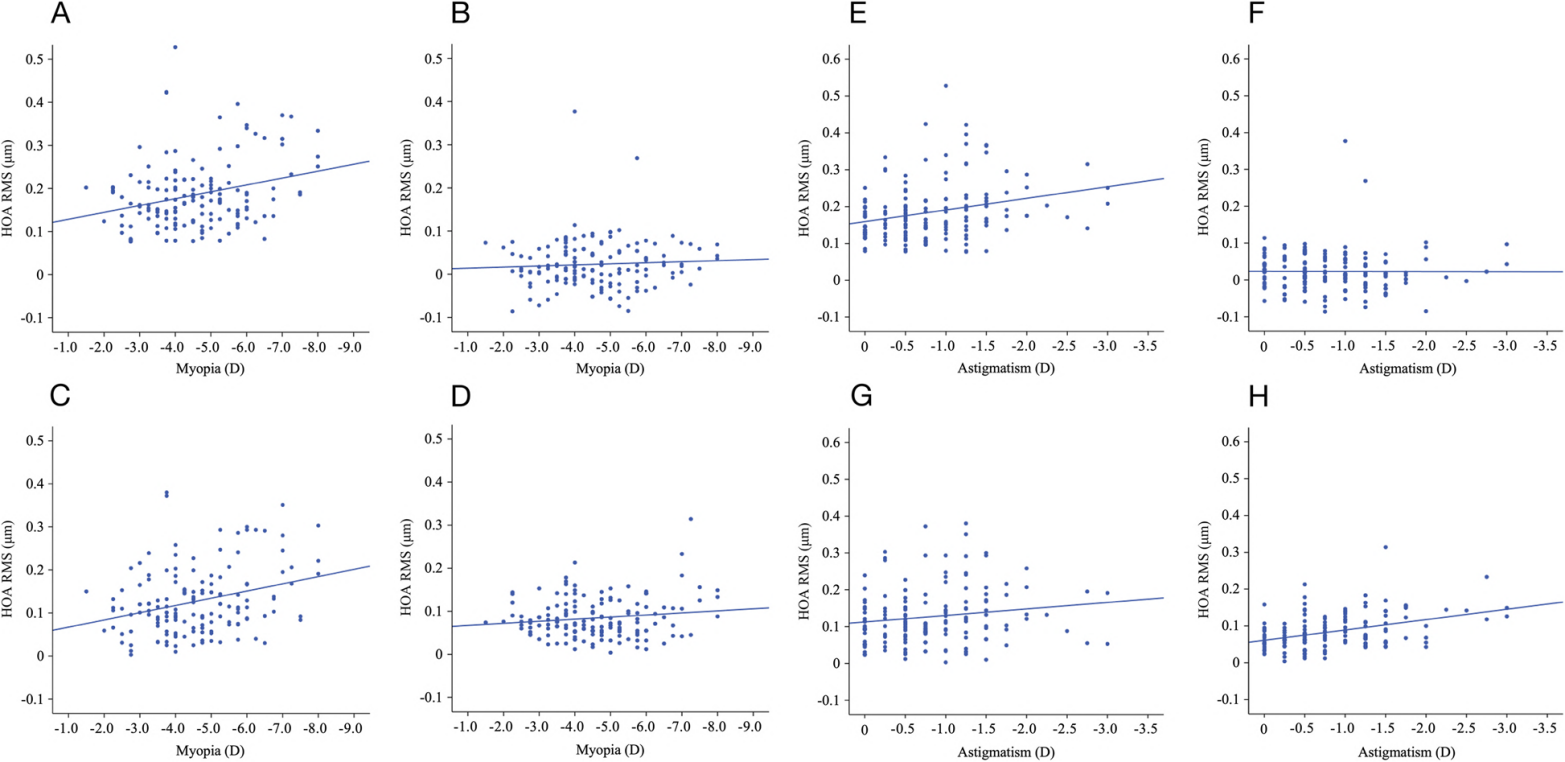
Analysis of the relationship between myopia and total eye HOA, astigmatism and total eye HOA. Linear regression was chosen for the analysis. **A **Myopia and total eye tHOA (*R*^2^ = 0.076, *P* < 0.001); **B **Myopia and total eye SA (*R*^2^ = 0.004, *P* = 0.453); **C **Myopia and total eye CA (*R*^2^ = 0.088, *P* < 0.001); **D **Myopia and total eye TA (*R*^2^ = 0.020, *P* = 0.084); **E **Astigmatism and total eye tHOA (*R*^2^ = 0.069, *P* = 0.001); **F **Astigmatism and total eye SA (*R*^2^ < 0.001, *P* = 0.959); **G **Astigmatism and total eye CA (*R*^2^ = 0.023, *P* = 0.065); **H **Astigmatism and total eye TA (*R*.^2^ = 0.158, *P* < 0.001)

Pearson’s correlation analysis showed that there was a significant positive correlation between the magnitude of myopia and total eye tHOA (*r* = -0.276, *P* = 0.001), total eye CA (*r* = -0.297, *P* < 0.001), corneal tHOA (*r* = -0.344, *P* < 0.001), corneal CA (*r* = -0.296, *P* < 0.001), corneal TA (*r* = -0.221, *P* = 0.007), internal tHOA (*r* = -0.201, *P* = 0.014), and ${{\text{Z}}}_{3}^{-3}$ (vertical TA, *r* = -0.238, *P* = 0.003) after SMILE, whereas a significant negative correlation was found between the magnitude of myopia and postoperative ${{\text{Z}}}_{3}^{-1}$(vertical CA, *r* = 0.291, *P* < 0.001). The magnitude of astigmatism was significantly positively correlated with total eye tHOA (*r* = -0.264, *P* = 0.001), total eye TA (*r* = -0.397, *P* < 0.001), corneal tHOA (*r* = -0.254, *P* = 0.002), corneal TA (*r* = -0.320, *P* < 0.001), internal tHOA (*r* = -0.237, *P* = 0.004), and ${{\text{Z}}}_{3}^{-3}$ (vertical TA, *r* = -0.198, *P* = 0.015) after SMILE. The specific correlations between myopia/astigmatism and HOA are shown in Table 3 and Fig. 2.

**Table 3:** Analysis of the relationship between myopia, astigmatism and HOA

Parameters	Myopia		Astigmatism	
	***r***	***P***	***r***	***P***
Total eye HOA RMS				
tHOA	-0.276	**0.001**	-0.264	**0.001**
SA	-0.062	0.453	-0.004	0.959
CA	-0.297	** < 0.001**	-0.151	0.065
TA	-0.142	0.084	-0.397	** < 0.001**
Cornea HOA RMS				
tHOA	-0.344	** < 0.001**	-0.254	**0.002**
SA	0.136	0.098	0.028	0.735
CA	-0.296	** < 0.001**	-0.072	0.381
TA	-0.221	**0.007**	-0.320	** < 0.001**
Internal HOA RMS				
tHOA	-0.201	**0.014**	-0.237	**0.004**
SA	-0.158	0.053	-0.021	0.801
CA	-0.150	0.068	-0.144	0.080
TA	-0.135	0.101	-0.102	0.214
Other total eye HOA RMS				
${{\text{Z}}}_{3}^{-3}$	-0.238	**0.003**	-0.198	**0.015**
${{\text{Z}}}_{3}^{-1}$	0.291	** < 0.001**	-0.029	0.724
${{\text{Z}}}_{3}^{1}$	-0.034	0.677	0.002	0.978
${{\text{Z}}}_{3}^{3}$	-0.063	0.445	-0.105	0.202
${{\text{Z}}}_{4}^{-4}$	-0.035	0.668	0.051	0.538
${{\text{Z}}}_{4}^{-2}$	-0.035	0.670	-0.039	0.638
${{\text{Z}}}_{4}^{2}$	-0.023	0.776	0.133	0.104
${{\text{Z}}}_{4}^{4}$	0.035	0.673	0.123	0.133

*CA* Coma aberration, *HOA* High order aberration, *RMS* Root mean square, *SA* Spherical aberration, *TA* Trefoil aberration, *tHOA* Total high order aberration; ${{\text{Z}}}_{3}^{-3}$ = vertical trifoil aberration; ${{\text{Z}}}_{3}^{-1}$ = vertical coma aberration; ${{\text{Z}}}_{3}^{1}$ = horizontal coma aberration; ${{\text{Z}}}_{3}^{3}$ = oblique trifoil aberration; ${{\text{Z}}}_{4}^{-4}$ = oblique quadrafoil aberration; ${{\text{Z}}}_{4}^{-2}$ = oblique secondary astigmatism; ${{\text{Z}}}_{4}^{2}$ = vertical secondary astigmatism; ${{\text{Z}}}_{4}^{4}$ = vertical quadrafoil aberration. All values are displayed in the form of mean ± standard deviation. *P* values < 0.05 at Pearson analysis are presented in boldface

### Correlations between myopia/astigmatism and HOA in different subgroups

**Fig. 3:**
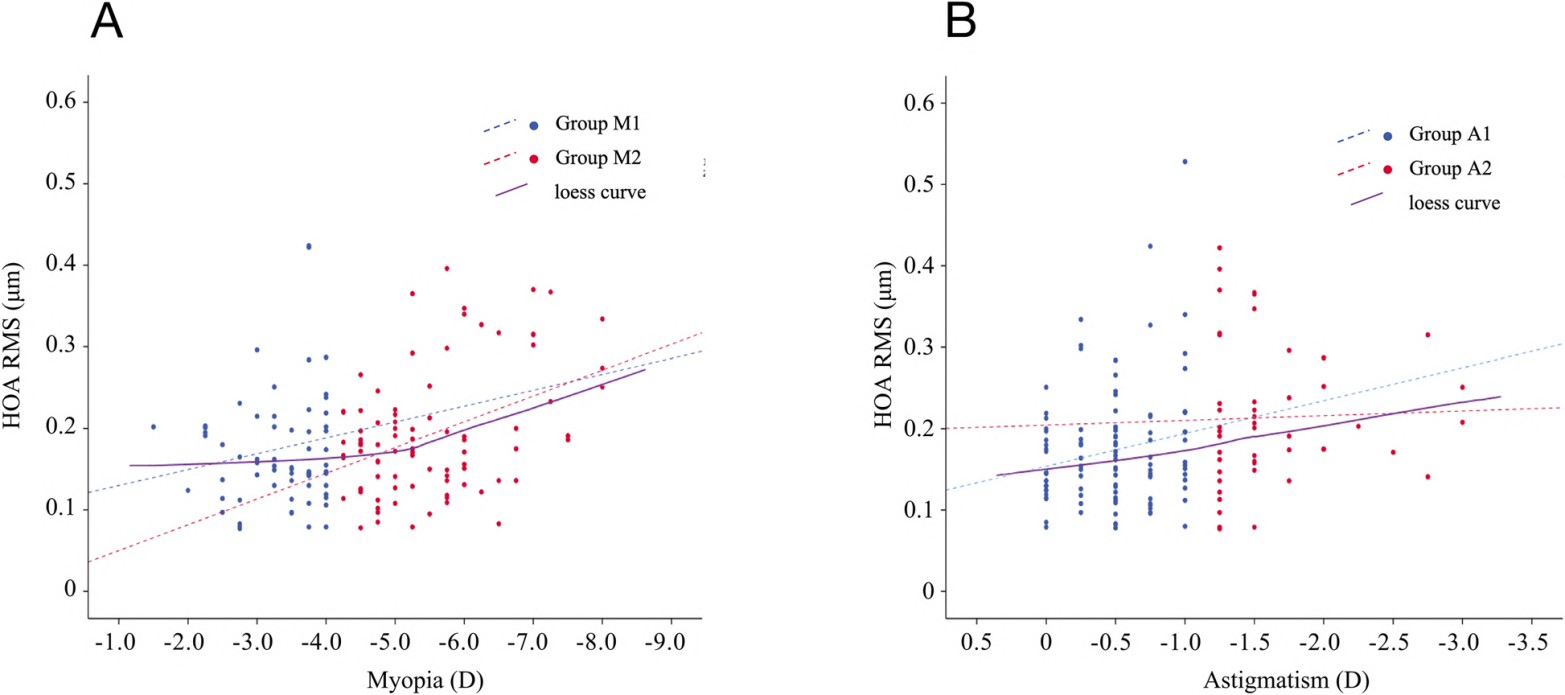
Analysis of the relationship between myopia/astigmatism and total eye tHOA under different groups. Linear regression and locally weighted regression (loess, Epanechnikov kernel) were chosen for the analysis. **A **Myopia and total eye tHOA (Group M1, *R*^2^ = 0.022, *P* = 0.240; Group M2, *R*^2^ = 0.163, *P* < 0.001); **B ** Astigmatism and total eye tHOA (Group A1, *R*^2^ = 0.037, *P* = 0.052; Group A2, *R*^2^ = 0.001, *P* = 0.829). The dashed line refers to the linear regression curve of the corresponding group, purple solid line refers to loess regression curve

The degree of myopia in Group M1 only had a significant positive correlation with ${{\text{Z}}}_{3}^{-3}$ (vertical TA, *r* = -0.262, *P* = 0.035), whereas the degree of myopia in Group M2 had a significant positive correlation with the total eye tHOA (*r* = -0.404, *P* < 0.001), total eye CA (*r* = -0.366, *P* = 0.001), total eye TA (*r *= -0.354, *P* = 0.001), corneal tHOA (*r* = -0.320, *P* = 0.003), corneal CA (*r* = -0.288, *P* = 0.008), corneal TA (*r* = -0.248, *P* = 0.022), and ${{\text{Z}}}_{3}^{-3}$ (vertical TA, *r* = -0.415, *P* < 0.001), and a significant negative correlation with ${{\text{Z}}}_{3}^{-1}$(vertical CA, *r* = 0.301, *P* = 0.005). In Group A1, the degree of astigmatism was significantly positively correlated only with the total eye TA (*r* = -0.297, *P* = 0.002), whereas in Group A2, the degree of astigmatism was significantly positively correlated with the total eye TA (*r* = -0.317, *P* = 0.030), corneal tHOA (*r* = -0.436, *P* = 0.002), corneal TA (*r* = -0.415, *P* = 0.004), internal tHOA (*r* = -0.414, *P* = 0.004), and internal TA (*r* = -0.353, *P* = 0.015). Specific correlations between the severity of myopia/astigmatism and HOA in the different subgroups are presented in Table 4 and Fig. 3.

**Table 4:** Analysis of the relationship between myopia/astigmatism and HOA under different groups

Parameters	Group M1		Group M2		Group A1		Group A2	
	***r***	***P***	***r***	***P***	***r***	***P***	***r***	***P***
Total eye HOA RMS								
tHOA	-0.148	0.240	-0.404	** < 0.001**	-0.192	0.052	-0.032	0.829
SA	-0.170	0.177	-0.114	0.299	-0.039	0.699	-0.109	0.465
CA	-0.093	0.459	-0.366	**0.001**	-0.096	0.336	0.101	0.499
TA	-0.079	0.530	-0.354	**0.001**	-0.297	**0.002**	-0.317	**0.030**
Cornea HOA RMS								
tHOA	0.020	0.875	-0.320	**0.003**	0.059	0.553	-0.436	**0.002**
SA	-0.049	0.701	0.064	0.562	0.074	0.459	0.214	0.148
CA	-0.027	0.830	-0.288	**0.008**	0.085	0.395	-0.128	0.390
TA	0.154	0.222	-0.248	**0.022**	-0.035	0.727	-0.415	**0.004**
Internal HOA RMS								
tHOA	-0.092	0.464	-0.104	0.344	-0.088	0.379	-0.414	**0.004**
SA	-0.099	0.435	-0.129	0.238	-0.093	0.351	-0.241	0.103
CA	-0.093	0.463	-0.117	0.288	-0.109	0.273	-0.144	0.334
TA	0.040	0.752	-0.109	0.319	0.167	0.091	-0.353	**0.015**
Other total eye HOA RMS								
${{\text{Z}}}_{3}^{-3}$	-0.262	**0.035**	-0.415	** < 0.001**	0.007	0.942	-0.117	0.435
${{\text{Z}}}_{3}^{-1}$	0.140	0.266	0.301	**0.005**	-0.081	0.414	-0.134	0.371
${{\text{Z}}}_{3}^{1}$	-0.084	0.507	-0.022	0.842	0.013	0.900	0.069	0.643
${{\text{Z}}}_{3}^{3}$	0.064	0.615	0.025	0.821	0.012	0.903	-0.021	0.889
${{\text{Z}}}_{4}^{-4}$	-0.039	0.755	-0.112	0.306	-0.063	0.526	0.152	0.309
${{\text{Z}}}_{4}^{-2}$	0.197	0.116	0.052	0.638	0.055	0.584	-0.032	0.831
${{\text{Z}}}_{4}^{2}$	-0.027	0.831	0.115	0.296	0.038	0.703	0.141	0.343
${{\text{Z}}}_{4}^{4}$	0.071	0.572	0.084	0.442	0.022	0.823	0.055	0.716

*CA* Coma aberration, *HOA* High order aberration, *RMS* Root mean square, *SA* Spherical aberration, *TA* Trefoil aberration, *tHOA* Total high order aberration; ${{\text{Z}}}_{3}^{-3}$ = vertical trifoil aberration; ${{\text{Z}}}_{3}^{-1}$ = vertical coma aberration; ${{\text{Z}}}_{3}^{1}$ = horizontal coma aberration; ${{\text{Z}}}_{3}^{3}$ = oblique trifoil aberration; ${{\text{Z}}}_{4}^{-4}$ = oblique quadrafoil aberration; ${{\text{Z}}}_{4}^{-2}$ = oblique secondary astigmatism; ${{\text{Z}}}_{4}^{2}$ = vertical secondary astigmatism; ${{\text{Z}}}_{4}^{4}$ = vertical quadrafoil aberration. All values are displayed in the form of mean ± standard deviation. *P* values < 0.05 at Pearson analysis are presented in boldface

### Relationship of other factors to HOA

**Fig. 4:**
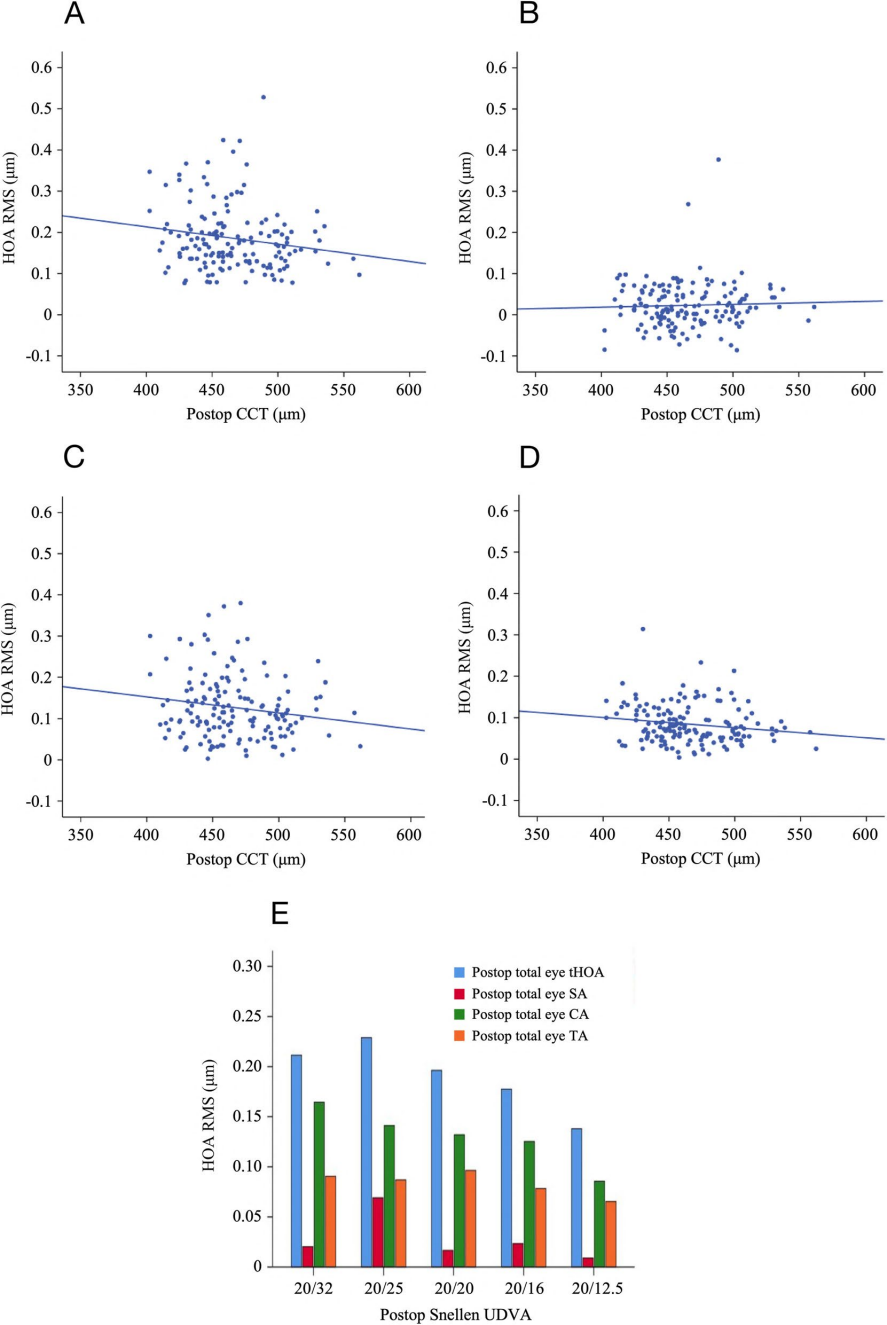
Analysis of the relationship between postop CCT, different postop UDVA and total eye HOA. Linear regression was chosen for the analysis. **A **Postop CCT and total eye tHOA (*R*^2^ = 0.029, *P* = 0.036); **B **Postop CCT and total eye SA (*R*^2^ = 0.001, *P* = 0.621); **C **Postop CCT and total eye CA (*R*^2^ = 0.026, *P* = 0.047); **D **Postop CCT and total eye TA (*R*^2^ = 0.029, *P* = 0.039); **E **The level of total eye HOA under different postop UDVA after SMILE

Significant positive correlations were found between corneal lenticule thickness and HOA including total eye tHOA (*r* = 0.342, *P* < 0.001), total eye CA (*r* = 0.314, *P* < 0.001), total eye TA (*r* = 0.283, *P* < 0.001), corneal tHOA (*r* = 0.396, *P* < 0.001), corneal CA (*r* = 0.281, *P* = 0.001), corneal TA (*r* = 0.318, *P* < 0.001), internal tHOA (*r* = 0.268, *P* = 0.001), internal CA (*r* = 0.186, *P* = 0.023), and ${{\text{Z}}}_{3}^{-3}$ (vertical TA, *r* = 0.283, *P* < 0.001). Additionally, there was a negative correlation between corneal lenticule thickness and ${{\text{Z}}}_{3}^{-1}$(vertical CA: *r* = -0.235, *P* = 0.004). Postoperative CCT was significantly negatively correlated with total eye tHOA (*r* = -0.171, *P* = 0.036), total eye CA (*r* = -0.162, *P* = 0.047), and total eye TA (*r* = -0.169, *P* = 0.039) and significantly positively correlated with ${{\text{Z}}}_{3}^{-1}$(vertical CA, *r* = 0.200, *P* = 0.014). There was a significant positive correlation between postoperative uncorrected distance visual acuity (UDVA) and total eye tHOA (*r* = 0.223, *P* = 0.006), total eye CA (*r* = 0.163, *P* = 0.046), and internal SA (*r* = 0.210, *P* = 0.010) and a significant negative correlation with corneal SA (*r* = -0.167, *P* = 0.041). Postoperative corneal Km and Cyl values were also correlated with most HOA (*P* < 0.05). However, other parameters, such as age, eyes, and postoperative IOP, were only correlated with specific HOA, and the correlations between specific factors and HOA are shown in Table 5 and Fig. 4.

**Table 5:** Analysis of the relationship between other factors and HOA

Parameters	Age		Eyes		Lenticule thickness		Postop CCT		Postop IOP		Postop sphere		Postop cylinder		Postop UDVA		Postop Km		Postop Cyl	
	***r***	***P***	***r***	***P***	***r***	***P***	***r***	***P***	***r***	***P***	***r***	***P***	***r***	***P***	***r***	***P***	***r***	***P***	***r***	***P***
Total eye HOA RMS																				
tHOA	-0.141	0.084	-0.081	0.324	0.342	** < 0.001**	-0.171	**0.036**	-0.013	0.877	-0.113	0.170	0.046	0.580	0.223	**0.006**	-0.066	0.528	0.375	** < 0.001**
SA	-0.007	0.928	-0.079	0.334	0.051	0.538	0.041	0.621	-0.047	0.565	0.052	0.529	0.052	0.528	0.104	0.207	-0.088	0.401	-0.027	0.793
CA	-0.120	0.145	-0.080	0.331	0.314	** < 0.001**	-0.162	**0.047**	-0.040	0.629	-0.119	0.148	0.024	0.767	0.163	**0.046**	-0.126	0.228	0.343	**0.001**
TA	-0.091	0.266	-0.001	0.994	0.283	** < 0.001**	-0.169	**0.039**	0.138	0.093	-0.016	0.843	-0.026	0.753	0.144	0.078	0.198	0.056	0.178	0.085
Cornea HOA RMS																				
tHOA	-0.182	**0.026**	-0.008	0.925	0.396	** < 0.001**	-0.118	0.150	-0.013	0.873	0.023	0.783	-0.002	0.984	0.069	0.401	-0.425	** < 0.001**	0.233	**0.024**
SA	-0.179	**0.028**	0.071	0.391	-0.127	0.123	0.101	0.219	-0.009	0.913	0.086	0.296	0.119	0.148	-0.167	**0.041**	0.182	0.079	-0.016	0.881
CA	-0.082	0.321	-0.005	0.949	0.281	**0.001**	-0.084	0.308	-0.026	0.751	-0.026	0.752	-0.046	0.575	0.044	0.595	-0.458	** < 0.001**	0.260	**0.011**
TA	-0.146	0.074	0.008	0.927	0.318	** < 0.001**	-0.089	0.281	0.041	0.618	0.101	0.219	-0.010	0.906	0.018	0.829	-0.100	0.336	-0.002	0.983
Internal HOA RMS																				
tHOA	-0.121	0.139	-0.008	0.927	0.268	**0.001**	-0.088	0.287	-0.012	0.883	0.004	0.960	0.056	0.492	0.130	0.114	-0.317	**0.002**	0.136	0.192
SA	0.148	0.071	-0.114	0.165	0.143	0.081	-0.058	0.481	-0.026	0.754	-0.038	0.648	-0.072	0.384	0.210	**0.010**	-0.221	**0.032**	-0.007	0.950
CA	-0.099	0.227	0.047	0.570	0.186	**0.023**	-0.032	0.702	0.004	0.961	0.054	0.514	0.038	0.644	0.051	0.537	-0.387	** < 0.001**	0.097	0.355
TA	-0.101	0.220	0.078	0.345	0.156	0.057	-0.073	0.373	0.017	0.833	0.033	0.692	-0.019	0.820	0.070	0.395	-0.032	0.759	0.023	0.825
Other total eye HOA RMS																				
${{\text{Z}}}_{3}^{-3}$	-0.092	0.262	-0.082	0.319	0.283	** < 0.001**	-0.039	0.638	0.050	0.543	-0.066	0.419	0.070	0.392	0.065	0.431	0.165	0.112	0.367	** < 0.001**
${{\text{Z}}}_{3}^{-1}$	0.049	0.553	0.153	0.062	-0.235	**0.004**	0.200	**0.014**	0.034	0.684	0.103	0.211	0.062	0.453	-0.093	0.256	0.036	0.729	-0.321	**0.002**
${{\text{Z}}}_{3}^{1}$	-0.105	0.199	0.038	0.645	0.028	0.732	0.041	0.620	-0.006	0.945	0.002	0.979	0.232	**0.004**	-0.033	0.685	0.011	0.916	0.129	0.216
${{\text{Z}}}_{3}^{3}$	-0.031	0.709	-0.432	** < 0.001**	0.096	0.241	-0.070	0.394	0.043	0.605	-0.022	0.791	-0.159	0.051	0.016	0.849	0.000	0.998	0.109	0.297
${{\text{Z}}}_{4}^{-4}$	0.082	0.320	-0.454	** < 0.001**	0.009	0.911	-0.058	0.478	-0.043	0.600	-0.021	0.802	-0.137	0.095	-0.040	0.624	0.047	0.650	-0.019	0.856
${{\text{Z}}}_{4}^{-2}$	0.046	0.578	-0.081	0.326	0.046	0.579	-0.044	0.592	0.185	**0.024**	-0.187	**0.022**	0.008	0.923	0.155	0.059	0.032	0.762	-0.211	**0.041**
${{\text{Z}}}_{4}^{2}$	0.147	0.073	-0.082	0.317	-0.035	0.673	-0.111	0.176	-0.133	0.105	-0.133	0.104	0.160	0.051	0.095	0.247	0.136	0.190	0.020	0.849
${{\text{Z}}}_{4}^{4}$	0.015	0.853	0.025	0.765	-0.080	0.331	0.071	0.388	0.027	0.740	0.060	0.469	-0.082	0.320	-0.075	0.364	-0.249	**0.016**	-0.300	**0.003**

*CA* Coma aberration, *CCT* Central corneal thickness, *Cyl* Cylindrical Lens, *HOA* High order aberration, *IOP* Intraocular pressure, *Km* Mean keratometry, *Postop* Postoperative, *RMS* Root mean square, *SA* Spherical aberration, *TA* Trefoil aberration, *tHOA* Total high order aberration, *UDVA* Uncorrected distance visual acuity. UDVA was statistically analyzed in the form of LogMAR; ${{\text{Z}}}_{3}^{-3}$ = vertical trifoil aberration; ${{\text{Z}}}_{3}^{-1}$ = vertical coma aberration; ${{\text{Z}}}_{3}^{1}$ = horizontal coma aberration; ${{\text{Z}}}_{3}^{3}$ = oblique trifoil aberration; ${{\text{Z}}}_{4}^{-4}$ = oblique quadrafoil aberration ${{\text{Z}}}_{4}^{-2}$ = oblique secondary astigmatism; ${{\text{Z}}}_{4}^{2}$ = vertical secondary astigmatism; ${{\text{Z}}}_{4}^{4}$ = vertical quadrafoil aberration. All values are displayed in the form of mean ± standard deviation. *P* values < 0.05 at Pearson analysis are presented in boldface

## Discussion

Most studies have shown a direct relationship between the degree of myopia and induction of HOA caused by SMILE [17–20]. Higher levels of myopia result in thicker lenticules being cut, reducing corneal thickness and stability, causing excessive corneal deformation and increasing HOA. At the same time, the greater the degree of ablation, the greater the refractive difference between the unablated and ablated areas, which also causes HOA induction [18]. Similar findings were observed in this study, but there were still some differences compared to other studies regarding different types of HOA induction. For example, Li et al. [21] observed that in high myopia, both SA and CA induction increased with myopia severity, although SA induction varied differently from the preoperative myopic and CA curves. They believed that the higher the degree of myopia, the thicker the lenticule that needs to be removed, which may cause significant changes in corneal anterior asphericity. Subsequently, Kwon et al. [22] proposed a prediction that corneal flattening may produce a large amount of ocular SA; however, it should be noted that there is a nonlinear relationship between SA induction and myopia, which may be related to differences in biomechanical properties at different depths of the cornea. Jin et al. [23] observed that SA increases in the anterior corneal surface and whole cornea after SMILE. Moreover, SA induction in the high myopia group was significantly higher than that in the mild to moderate myopia group, while the increase in vertical CA also significantly exceeded that in the mild to moderate myopia group, which was consistent with the findings of Chen and Wu et al.[17, 18], and the pattern of change between vertical CA and myopia was consistent with the findings of this study. However, this study did not observe an increase in the postoperative SA with increasing degrees of myopia. The results could be affected by the selection of smaller pupil diameters or the lack of comparison of differences in HOA before and after surgery; therefore, the results may be influenced by the impact of myopia on HOA (some studies have shown that HOA is higher in myopic eyes than in other refractive states [24, 25]). It can be speculated that myopia severity likely influences the level of HOA after surgery. The increase in HOA was associated with the deterioration of corneal biomechanical stability and flatness, whereas the increase in CA may be due to the increase in ablation eccentricity caused by the increase in myopia.(Studies on HOA after SMILE are summarized in Supplementary Table 1).

Some researchers have studied the relationship between the degree of astigmatism and induction of HOA, but no consensus [26–28]. Zhong et al. [15] observed that there was no significant difference in the induction of tHOA, SA, or CA between high and low astigmatism groups, which is consistent with the findings of Liu et al. [29] and Jun et al. [27] However, Huang et al. [28] confirmed that in eyes with large ablation eccentricity, the values of induced CA and SA were higher in the high astigmatism group than in the low astigmatism group, which was similar to the results of this study. Huang et al. further pointed out that for patients with high astigmatism, when the eccentricity of fixation exceeds 0.20 mm, the sensitivity between the significant induction of CA and the eccentric distance after SMILE increased significantly [28]. Subsequently, Lee et al. [30] observed that in cases where the eccentric distance of fixation exceeded 0.335 mm, aberrations exhibited similar significant differences. Therefore, if the ablation eccentricity exceeds a reasonable range, there will be a significant correlation between eccentricity and induced aberrations. However, other studies have reached conclusions inconsistent with the views of this study. Ding et al. [31] confirmed that regardless of the astigmatism intensity, there was no direct relationship between induced aberrations and optical zone eccentricity. Liu et al. [32] found that SA induction in the high astigmatism group was smaller than that in the non-astigmatism group, which they speculated was related to its larger functional optical zone (FOZ). The size of the FOZ may affect the effect of fixation eccentricity on induced CA, as the shape of the FOZ in high astigmatism is elliptical and has a different axis [30]. It can be understood that a higher astigmatism correction results in a larger FOZ, and the increased FOZ reduces the induction of SA and mitigates the induction of CA by eccentricity. The presence of eccentricity and counteracting effects brought about by the optical zone ultimately merge to form the degree of induction of HOA; thus, the degree of astigmatism was not significantly correlated with the induction of SA and CA. Although this study still found a link between postoperative tHOA, TA, and the degree of astigmatism, this may be due to postoperative corneal irregularities or TA inherent in astigmatism [33]. The lack of a link between postoperative SA and CA, and the degree of astigmatism in this study may be explained by the FOZ theory described above.

Total eye and corneal HOA showed more consistent behavior in most analyses, although internal HOA did not exhibit a significant association with parameters, such as myopia and astigmatism, in the majority of cases. This implies that the internal HOA is relatively stable, which may be another HOA compensation mechanism besides the posterior surface of the cornea [34]. Meanwhile, the analysis of internal HOA in this study was more consistent with the analysis of the posterior corneal surface in the study by Jin et al. [20]. They found that the HOA of the posterior corneal surface did not change with the degree of myopia and that it was stable in compensating for HOA before and after SMILE.

Further subgroup analysis revealed that there was almost no significant correlation between postoperative HOA (only vertical TA) and low myopia, but the opposite was true when moderate to high myopia was corrected. This seems to reflect that myopia is more likely to cause higher postoperative HOA when corrected to a higher degree. However, correction for low myopia is relatively safe. As shown in Fig. 3, the degree of myopia correction and the increase in postoperative HOA occured at a certain “node”, which may be due to the difficulty of compensating for internal HOA caused by higher degrees of myopia correction, or it may be related to corneal biomechanical stability: when correction leads to a thin residual corneal stroma bed thickness, reaching a critical value, it may be difficult to withstand IOP, which may be reflected preferentially in HOA [35]. Astigmatism was significantly associated with postoperative tHOA in both moderate/high and low astigmatism, although the former was associated with more types of HOA in addition to tHOA. The mechanism for this difference is different from myopia, and the increase in postoperative HOA may be related to a significant decrease in cutting eccentricity and corneal unevenness due to the correction of a higher degree of astigmatism. However, the highest degree of astigmatism also differed more from the degree of myopia in this study (the highest astigmatism degree was only -3D). Figure 3 shows that the fitting curve between the degree of astigmatism and postoperative tHOA has an approximately linear relationship. This may imply that there are differences in the mechanisms by which astigmatism affects SMILE, as opposed to myopia. However, correcting higher degrees of astigmatism does indeed lead to more HOA, which suggests that correcting higher degrees of astigmatism is less safe than correcting lower astigmatism.

Postoperative corneal lenticule thickness, postoperative UDVA, postoperative Cyl, postoperative Km, and postoperative CCT were all variables that have been shown to correlate with postoperative HOA. Results from the studies by Feng et al. [36] and Hu et al. [37] indicated a strong relationship between postoperative CCT and HOA after laser-assisted in situ keratomileusis (LASIK). They speculated that this could be related to worse corneal biomechanical properties after surgery, but they also believed that HOA induced by preoperative CCT could affect postoperative HOA. As some researchers have argued otherwise, more studies were needed to determine whether CCT and HOA are correlated [38, 39]. Decreased visual quality was generally associated with increased HOA, as Miller et al. [40] found that decreased BCVA was associated with higher levels of SA, but not CA. Namba et al. [41] reported an association between increased HOA and deterioration in visual function, which was similar to the results of this study. The increases in tHOA, CA, and SA should all decrease visual acuity, although the association between postoperative SA and HOA may not have been evident in this study because of pupillary factors. Other parameters, such as lenticule thickness and postoperative Km and Cyl, were determined based on the degree of myopia and astigmatism. In addition, this study did not find many associations between postoperative HOA and age, postoperative IOP, eyes, postoperative DS, or DC. Although some scholars have proposed an association between age, IOP, and gender with HOA [37, 42, 43], further research is needed to investigate its impact on introducing HOA after SMILE.

This study was the first to examine the changes in HOA after SMILE for myopia and astigmatism as well as to examine the factors associated with postoperative HOA. However, this study has some limitations. First, this study examined the pupil diameter (4 mm) under normal light conditions. Since a larger pupil diameter is believed to be associated with a higher HOA, future studies can broaden the investigation of pupil size to investigate the variations in HOA under dark conditions. Second, for bilaterally treated patients, there may be a correlation between the two eyes of one patient, which is a common mistake in ophthalmology research because the overall variance of a sample of measurements from both eyes is likely to be underestimated, increasing the risk of a type 1 error [44]. Finally, since this study focused on the association between postoperative factors and HOA, factors such as the focusing method and astigmatism axis adjustment that would significantly affect postoperative HOA were not explored [45, 46].

## Conclusion

This study showed that an increase in the degree of myopia and astigmatism was associated with a higher degree of postoperative HOA after SMILE; however, the increase in HOA was not linearly correlated with the increase in myopia. Although there was a critical value, the degree of astigmatism showed a similar linear correlation. This finding suggested that SMILE can maintain excellent postoperative visual experience, at least in the case of mild myopia. The level of HOA after SMILE was associated with many factors; however, its specific clinical significance requires further specialized research. Therefore, postoperative HOA was affected by multiple factors, and more research on HOA induction and its causes in the future will be able to further explore the relevant findings of this study.

### Supplementary Information

Supplementary Material 1. 

## Data Availability

The data have not been placed in any online data storage. The datasets generated and analyzed during the study are available upon request from the first author.

## Abbreviations

CDVA: Corrected distance visual acuity
CA: Coma aberration
CCT: Central corneal thickness
FOZ: Functional optical zone
FS-LASIK: Femtosecond laserassisted LASIK
HOA: Higher-order aberration
IOP: Intraocular pressure
LASEK: Laser-assisted subepithelial keratomileusis
LASIK: Laser-assisted in situ keratomileusis
OCT: Optical coherence tomography
PRK: Photorefractive keratectomy
RCST/CT: Residual corneal stromal thickness/corneal thickness
RMS: Root mean square
RK: Radial keratotomy
SA: Spherical aberration
SMILE: Small incision lenticule extraction
tHOA: Total higher-order aberration
TA: Trefoil aberration
UDVA: Uncorrected distance visual acuity
