# Supplementary material for: Association of myopia and astigmatism with postoperative ocular high order aberration after small incision lenticule extraction
Source: BMC Ophthalmol. 2024 May 13;24:211. doi: 10.1186/s12886-024-03475-w (PMC11089751; doi:10.1186/s12886-024-03475-w)
Supplement: Supplementary file 1 — Supplementary Material 1. [file 12886_2024_3475_MOESM1_ESM.pdf]

Supplementary Table 1 Summary of studies involving HOA after SMILE and related parameters

Study	Year	Country	Eyes(n)	Mean age	Mean Sphere(D)	Mean Cylinder(D)	Preop CCT(μm)	Preop CDVA(logMAR)	Follow-up (mo)	Aberration analyzer	Position	Surgery-related parameters(Model; Energy; Cap Thickness/Diameter; Lenticule Diameter; Incision Length/Location; Other technologies)	Postop tHOA	Postop SA	Postop CA	Postop TA
Xiaojing Li [1]	2015	China	55	22.2±3.0	-5.74±1.39	-0.66±0.70	/	0.00	6	Pentacam HR	Cornea	500-kHz Visumax system; 110-120nJ; 110μm/7mm; 6.0mm; 2.0-5.0mm/90°; -	0.95±0.23	0.41±0.18	V -0.61±0.39<br>H -0.24±0.19	/
Minjie Ye [2]	2016	China	170	25.3±4.2	-5.03±1.89*	/	/	/	6	Pentacam HR	Cornea	500-kHz Visumax system; 130nJ; 120-130μm/-; 6.0mm; 4.0-5.0mm/90°; -	0.22±0.06	0.38±0.14	V -0.27±0.22<br>H 0.09±0.22	/
Wenjing Wu [3]	2016	China	73	24.5±6.3	-5.44±1.30	-0.71±0.67	551.5±24.6	/	3	Pentacam HR	Cornea	500-kHz Visumax system; -; 110μm/-; 6.0-6.5mm; 2.0-5.0mm/90°; -	0.82±0.24	0.48±0.17	0.54±0.25	0.10±0.06
Xiaoqin Chen [4]	2017	China	39	22.0±4.0	-4.41±1.23	-2.26±0.73	/	0.01±0.03	3	WaveScan	Whole eye	500-kHz Visumax system; 170nJ; 110μm/-; 6.0-6.5mm; 2.0-5.0mm/90°; -	0.27±0.07	0.09±0.05	/	/
Liuyang Li [5]	2017	China	19	24.6±4.7	-2.24±0.67	-0.66±0.60	550.8±33.0	/	3	WaveScan	Whole eye	500-kHz Visumax system; 155nJ; 110-120μm/7.5-7.6mm; 6.5mm; 3.00-3.13mm/90°; -	0.40±0.03	0.10±0.03	V -0.08±0.05<br>H -0.05±0.03	/
	2017	China	20	22.3±5.2	-4.26±0.57	-0.46±0.41	548.6±22.2	/	3	WaveScan	Whole eye	500-kHz Visumax system; 155nJ; 110-120μm/7.5-7.6mm; 6.5mm; 3.00-3.13mm/90°; -	0.46±0.02	0.22±0.03	V -0.18±0.03<br>H -0.19±0.03	/
	2017	China	21	24.1±4.9	-7.02±0.67	-0.67±0.64	552.9±28.2	/	3	WaveScan	Whole eye	500-kHz Visumax system; 155nJ; 110-120μm/7.5-7.6mm; 6.5mm; 3.00-3.13mm/90°; -	0.54±0.03	0.22±0.02	V -0.24±0.04<br>H -0.22±0.04	/

Hun Lee [6]	2018	South Korea	81	28.3±6.0	-4.66±1.25	-1.03±0.75	548.9±20.7	/	6	Pentacam HR	Cornea	500-kHz Visumax system; 100-115nJ; 120-140μm/-; 6.2-7.0mm; 2.0mm/-; Triple Centration Technique	0.61±0.18	0.37±0.13	0.35±0.19	/
Ikhyun Jun [7]	2018	South Korea	45	24.8±4.6	-3.19±1.55	-2.90±0.42	560.5±29.2	-0.05±0.07	6	Keratron Scout	Cornea	500-kHz Visumax system; -; -/-; -; -/-; Triple Centration Technique	0.56±0.14	0.24±0.13	0.31±0.15	0.22±0.13
HongYing Jin [8]	2018	China	65	24.5±7.3	-6.94±1.00	-0.76±0.68	552.6±25.6	/	3	Pentacam HR	Cornea	500-kHz Visumax system; 155nJ; 120μm/7.5-7.6mm; -; 2.0mm/120°; -	0.99±0.28	0.42±0.14	V -0.68±0.31<br>H -0.04±0.40	/
	2018	China	132	23.8±5.9	-4.13±1.00	-0.64±0.50	541.5±28.0	/	3	Pentacam HR	Cornea	500-kHz Visumax system; 155nJ; 120μm/7.5-7.6mm; -; 2.0mm/120°; -	0.66±0.20	0.26±0.13	V -0.36±0.27<br>H -0.06±0.27	/
David Sung Yong Kang [9]	2018	South Korea	55	28.6±6.4	-4.41±1.74	-0.90±0.66	553.3±26.1	0.01±0.01	3	Keratron Scout	Cornea	500-kHz Visumax system; -; -/-; -; -/-; -	0.57±0.18	0.34±0.14	0.35±0.18	/
	2018	South Korea	55	27.5±6.2	-3.79±1.52	-1.09±0.97	552.6±31.5	0.01±0.01	3	Keratron Scout	Cornea	500-kHz Visumax system; -; -/-; -; -/-; Triple Centration Technique	0.55±0.14	0.30±0.13	0.31±0.16	/
Manrong Yu [10]	2019	China	32	24.2±4.5	-4.10±0.90*	/	553.0±27.2	/	3	WASCA	Cornea	500-kHz Visumax system; 130nJ; 120μm/7.5mm; 6.5mm; 2.0mm/90°; -	0.39±0.13	-0.26±0.28	0.73±0.48	0.33±0.20
Meiyan Li [11]	2019	China	68	29.5±5.8	-5.95±1.37	-0.84±0.78	551.8±30.0	-0.07±0.11	3	Pentacam HR	Cornea	500-kHz Visumax system; 130nJ; 100μm/-; 6.5-6.8mm; 2.0-4.5mm/90°; -	0.81±0.25	0.42±0.12	0.59±0.28	/
Weiming Yang [12]	2019	China	29	26.5±7.5	-8.23±0.56	-0.94±0.71	542.9±17.4	-0.01±0.06	6	WASCA	Whole eye	500-kHz Visumax system; 130nJ; 110μm/7.5mm; 6.0-6.5mm; 2.0-2.5mm; -	0.55±0.10	0.28±1.00	0.42±0.14	0.12±0.07

Bing Qin [13]	2019	China	30	24.5±6.8	-6.40±1.29*	-0.79±0.60	540.5±30.1	0.02±0.05	6	WASCA	Cornea	500-kHz Visumax system; 130nJ; 110-120μm/-; 6.0-6.6mm; 2.0mm/90°; -	0.36±0.11	0.09±0.07	0.26±0.13	0.13±0.07
	2019	China	30	26.9±6.1	-10.06±0.77*	-1.24±1.05	547.2±25.1	0.00±0.05	6	WASCA	Cornea	500-kHz Visumax system; 130nJ; 110-120μm/-; 6.0-6.6mm; 2.0mm/90°; -	0.41±0.13	0.20±0.12	0.25±0.14	0.11±0.07
Jay Jiyong Kwak [14]	2020	South Korea	57	25.6±5.5	-4.37±1.98	-1.14±0.82	/	-0.04±0.04	3	iTrace	Cornea	500-kHz Visumax system; -; -/-; -; -/-; Triple Centration Technique	0.46±0.22	0.21±0.14	0.28±0.17	0.17±0.12
Mehmet Gulmez [15]	2020	Turkey	94	28.0±6.4	-4.89±2.31	-1.64±1.45	535.6±27.7	0.02±0.04	6	WaveLight	Cornea	500-kHz Visumax system; 150nJ; 130μm/-; 6.0-6.5mm , 2.0mm/135°; -	0.28±0.11	0.24±0.05	0.17±0.03	0.14±0.02
Yuanyuan Zhong [16]	2020	China	43	24.6±3.9	-5.12±1.77	-2.47±0.54	/	-0.03±0.04	48	Pentacam HR	Cornea	500-kHz Visumax system; 130nJ; 120μm/7.6mm; 6.0-6.9mm; 2.0mm/90°; -	0.84±0.29	0.30±0.17	0.61±0.30	/
	2020	China	31	25.3±4.2	-5.58±1.78	-0.55±0.28	/	-0.04±0.04	48	Pentacam HR	Cornea	500-kHz Visumax system; 130nJ; 120μm/7.6μm; 6.0-6.9mm; 2.0mm/90°; -	0.86±0.28	0.40±0.18	0.63±0.32	/
Fei Xia [17]	2020	China	26	28.3±7.8	-5.95±1.14	-0.76±0.48	544.1±28.2	/	1	Pentacam HR	Cornea	500-kHz Visumax system; 130nJ; 100-110μm/7.5-7.8mm; 6.5-6.8mm; 4.5mm/90°; -	0.49±0.25	0.14±0.09	V 0.05±0.07<br>H 0.07±0.09	/
Ruoyan Wei [18]	2020	China	103	28.7±5.0	-7.39±0.79	-0.93±0.69	549.4±29.1	-0.03±0.04	6	WASCA	Whole eye	500-kHz Visumax system; 130nJ; 110-120μm/-; 6.2-6.5mm; 2.0mm/90°; -	0.37±0.11	0.14±0.09	0.25±0.13	0.12±0.07
Ikhyun Jun [19]	2021	South Korea	91	27.8±6.0	-3.18±1.28	-0.93±0.69	565.2±24.4	-0.12±0.06	6	WaveLight	Cornea	500-kHz Visumax system; -; -/-; -; -/-; Triple Centration Technique	0.48±0.13	0.26±0.10	0.26±0.14	0.17±0.09

Ikhyun Jun	2021	South Korea	59	27.3±7.0	-3.26±1.49	-0.97±0.94	563.9±22.0	-0.11±0.07	6	WaveLight	Cornea	500-kHz Visumax system; -; -/-; -; -/-; Triple Centration Technique	0.53±0.16	0.34±0.13	0.29±0.17	0.15±0.09
D. Rex Hamilton [20]	2021	United States	49	31.6±6.3	-3.77±1.60	-0.50±0.46	/	/	1	Galilei G4	Cornea	500-kHz Visumax system; 125-130nJ(4.5 spot spacing); 120μm/7.5μm; 6.5mm; -/-; -	0.58±0.15	0.32±0.12	0.37±0.14	/
	2021	United States	49	29.1±5.1	-4.02±1.39	-0.10±0.19	/	/	1	Galilei G4	Cornea	500-kHz Visumax system; 125nJ(3.0 spot spacing); 120μm/7.5μm; 6.5mm; -/-; -	0.63±0.13	0.40±0.10	0.39±0.15	/
Yewei Yin [21]	2021	China	51	23.9±4.8	-7.96±0.94*	-0.79±0.51	/	/	6	iTrace	Whole eye	500-kHz Visumax system; 130nJ; 100-120μm/-; 6.0-6.8mm; 4.0mm/90°; -	0.59±0.60	0.26±0.45	0.40±0.41	0.16±0.12
Meng Lin [22]	2021	China	64	23.3±5.0	-4.17±1.55	-0.66±0.54	555.8±31.7	-0.02±0.04	6	OPD-Scan II	Whole eye	500-kHz Visumax system; 135nJ; 120μm/7-8mm; 6.4-6.9mm; 2.0mm/90°; -	0.53±0.26	0.14±0.10	0.29±0.17	0.29±0.11
Yu Zhang [23]	2022	China	102	28.2±6.1	-5.09±1.26	-0.63±0.31	548.0±24.0	/	6	WaveLight	Cornea	500-kHz Visumax system; 140nJ; 120μm/7.6μm; 6.5mm; 2.0mm/-; -	0.70±0.20	0.45±0.16	0.40±0.20	/
Mengjun Fu [24]	2022	China	36	29.4±5.9	-2.58±0.59	0.73±0.44	/	/	24	WASCA	Whole eye	500-kHz Visumax system; 130nJ; 120μm/-; 6.5-6.8mm; 2.0mm/90°; -	0.82±0.53	0.12±0.10	V -0.10±0.18<br>H 0.08±0.25	/
Cong Zhou [25]	2023	China	100	21.00#	-3.78±0.10	-3.61±0.10	541.1±3.5	0.00±0.00	3	Sirius	Cornea	500-kHz Visumax system; 130nJ; 110-120μm/-; 6.5mm; -/-; -	0.68±0.02	0.37±0.01	0.44±0.02	0.21±0.01
	2023	China	100	18.00#	-0.64±0.06	-0.58±0.06	546.9±3.5	0.00±0.00	3	Sirius	Cornea	500-kHz Visumax system; 130nJ; 110-120μm/-; 6.8mm; -/-; -	0.61±0.02	0.31±0.01	0.37±0.02	0.20±0.01

Wuqiang Luo [26]	2023	China	32	27.7±5.6	-7.84±0.64	-1.00±0.45	546.1±23.3	/	48	WASCA	Whole eye	500-kHz Visumax system; 130nJ; 110-120μm/-; 6.0-6.7mm; -/-; -	0.20±0.08	-3.87±2.43	2.88±2.14	/
This study	2024	China	65	28.6±4.9	-3.35±0.62	-0.76±0.60	537.5±29.7	-0.04±0.04	3	iTrace	Whole eye	500-kHz Visumax system; 110-120nJ; 110-120μm/7.6mm; 6.5mm; 3.0mm/120°; -	0.18±0.08	0.02±0.06	0.11±0.08	0.09±0.04
	2024	China	85	29.0±6.2	-5.53±0.99	-0.89±0.71	535.4±26.4	-0.03±0.04	3	iTrace	Whole eye	500-kHz Visumax system; 110-120nJ; 110-120μm/7.6mm; 6.5mm; 3.0mm/120°; -	0.19±0.77	0.02±0.05	0.14±0.08	0.08±0.05
	2024	China	103	29.3±5.7	-4.50±1.24	-0.47±0.35	532.9±25.0	-0.04±0.04	3	iTrace	Whole eye	500-kHz Visumax system; 110-120nJ; 110-120μm/7.6mm; 6.5mm; 3.0mm/120°; -	0.17±0.07	0.03±0.06	0.12±0.07	0.08±0.04
	2024	China	47	27.6±5.6	-4.78±1.64	-1.62±0.49	539.9±32.2	-0.03±0.04	3	iTrace	Whole eye	500-kHz Visumax system; 110-120nJ; 110-120μm/7.6mm; 6.5mm; 3.0mm/120°; -	0.21±0.09	0.02±0.06	0.15±0.09	0.10±0.06

* Spherical equivalent; # Median of values; CA = coma aberration; CCT = central corneal thickness; CDVA = corrected distance visual acuity; H = horizontal coma aberration; HOA = high order aberration; Postop = Postoperative; SA = spherical aberration; TA = trefoil aberration; tHOA = total high order aberration; V = vertical coma aberration. CDVA was statistically analyzed in the form of LogMAR. All values are displayed in the form of mean ± standard deviation.

References

[1] Li X, Wang Y, Dou R. Aberration compensation between anterior and posterior corneal surfaces after Small incision lenticule extraction and Femtosecond laser-assisted laser in-situ keratomileusis. Ophthalmic Physiol Opt. 2015;35(5):540-51.

[2] Ye MJ, Liu CY, Liao RF, Gu ZY, Zhao BY, Liao Y. SMILE and Wavefront-Guided LASIK Out-Compete Other Refractive Surgeries in Ameliorating the Induction of High-Order Aberrations in Anterior Corneal Surface. J Ophthalmol. 2016;2016:8702162.

[3] Wu W, Wang Y. Corneal Higher-Order Aberrations of the Anterior Surface, Posterior Surface, and Total Cornea After SMILE, FS-LASIK, and FLEx Surgeries. Eye Contact Lens. 2016;42(6):358-65.

[4] Chen X, Wang Y, Zhang J, Yang SN, Li X, Zhang L. Comparison of ocular higher-order aberrations after SMILE and Wavefront-guided Femtosecond LASIK for myopia. BMC Ophthalmol. 2017;17(1):42.

[5] Li L, Cheng GPM, Ng ALK, Chan TCY, Jhanji V, Wang Y. Influence of Refractive Status on the Higher-Order Aberration Pattern After Small Incision Lenticule Extraction Surgery. Cornea. 2017;36(8):967-72.

[6] Lee H, Yong Kang DS, Reinstein DZ, Arba-Mosquera S, Kim EK, Seo KY, Kim TI. Comparing corneal higher-order aberrations in corneal wavefront-guided transepithelial photorefractive keratectomy versus small-incision lenticule extraction. J Cataract Refract Surg. 2018;44(6):725-33.

[7] Jun I, Kang DSY, Reinstein DZ, Arba-Mosquera S, Archer TJ, Seo KY, Kim TI. Clinical Outcomes of SMILE With a Triple Centration Technique and Corneal Wavefront-Guided Transepithelial PRK in High Astigmatism. J Refract Surg. 2018;34(3):156-63.

[8] Jin HY, Wan T, Yu XN, Wu F, Yao K. Corneal higher-order aberrations of the anterior surface, posterior surface, and total cornea after small incision lenticule extraction (SMILE): high myopia versus mild to moderate myopia. BMC Ophthalmol. 2018;18(1):295.

[9] Kang DSY, Lee H, Reinstein DZ, Roberts CJ, Arba-Mosquera S, Archer TJ, Kim EK, Seo KY, Kim TI. Comparison of the Distribution of Lenticule Decentration Following SMILE by Subjective Patient Fixation or Triple Marking Centration. J Refract Surg. 2018;34(7):446-52.

[10] Yu M, Chen M, Liu W, Dai J. Comparative study of wave-front aberration and corneal Asphericity after SMILE and LASEK for myopia: a short and long term study. BMC Ophthalmol. 2019;19(1):80.

[11] Li M, Li M, Chen Y, Miao H, Yang D, Ni K, Zhou X. Five-year results of small incision lenticule extraction (SMILE) and femtosecond laser LASIK (FS-LASIK) for myopia. Acta Ophthalmol. 2019;97(3):e373-80.

[12] Yang W, Liu S, Li M, Shen Y, Zhou X. Visual Outcomes after Small Incision Lenticule Extraction and Femtosecond Laser-Assisted LASIK for High Myopia. Ophthalmic Res. 2020;63(4):427-33.

[13] Qin B, Zhao J, Li M, Yao P, Zhou X. The comparison of visual outcomes, aberrations, and Bowman's layer micro-distortions after femtosecond laser small-incision lenticule extraction (SMILE) for the correction of high and moderate myopia and myopic astigmatism. BMC Ophthalmol. 2019;19(1):138.

[14] Kwak JJ, Jun I, Kim EK, Seo KY, Kim TI. Clinical Outcomes of Small Incision Lenticule Extraction in Myopia: Study of Vector Parameters and Corneal Aberrations. Korean J Ophthalmol. 2020;34(1):76-84.

[15] Gulmez M, Tekce A, Kamıs U. Comparison of refractive outcomes and high-order aberrations after small incision lenticule extraction and wavefront-guided femtosecond-assisted laser in situ keratomileusis for correcting high myopia and myopic astigmatism. Int Ophthalmol. 2020;40(12):3481-9.

[16] Zhong Y, Li M, Han T, Fu D, Zhou X. Four-year outcomes of small incision lenticule extraction (SMILE) to correct high myopic astigmatism. Br J Ophthalmol. 2021;105(1):27-31.

[17] Xia F, Shen Y, Han T, Zhao J, Xu H, Zhou X. Small Incision Lenticule Extraction (SMILE) for Moderate and High Myopia: Seven-Year Outcomes of Refraction, Corneal Tomography, and Wavefront Aberrations. J Ophthalmol. 2020;2020:3825864.

[18] Wei R, Li M, Zhang H, Aruma A, Miao H, Wang X, Zhou J, Zhou X. Comparison of objective and subjective visual quality early after implantable collamer lens V4c (ICL V4c) and small incision lenticule extraction (SMILE) for high myopia correction. Acta Ophthalmol. 2020;98(8):e943-50.

[19] Jun I, Kang DSY, Roberts CJ, Lee H, Jean SK, Kim EK, Seo KY, Kim TI. Comparison of Clinical and Biomechanical Outcomes of Small Incision Lenticule Extraction With 120- and 140-μm Cap Thickness. Transl Vis Sci Technol. 2021;10(8):15.

[20] Hamilton DR, Chen AC, Khorrami R, Nutkiewicz M, Nejad M. Comparison of early visual outcomes after low-energy SMILE, high-energy SMILE, and LASIK for myopia and myopic astigmatism in the United States. J Cataract Refract Surg. 2021;47(1):18-26.

[21] Yin Y, Lu Y, Xiang A, Fu Y, Zhao Y, Li Y, Hu T, Du K, Hu S, Fu Q, Wu X, Wen D. Comparison of the optical quality after SMILE and FS-LASIK for high myopia by OQAS and iTrace analyzer: a one-year retrospective study. BMC Ophthalmol. 2021;21(1):292.

[22] Lin M, Zhou H, Hu Z, Huang J, Lu F, Hu L. Comparison of small incision lenticule extraction and transepithelial photorefractive keratectomy in terms of visual quality in myopia patients. Acta Ophthalmol. 2021;99(8):e1289-96.

[23] Zhang Y, Sun X, Chen Y. Comparison of Corneal Optical Quality After SMILE, Wavefront-Optimized LASIK and Topography-Guided LASIK for Myopia and Myopic Astigmatism. Front Med (Lausanne). 2022;9:870330.

[24] Fu M, Li M, Xian Y, Yu Z, Zhang H, Choi J, Niu L, Wang X, Zhou X. Two-Year Visual Outcomes of Evolution Implantable Collamer Lens and Small Incision Lenticule Extraction for the Correction of Low Myopia. Front Med (Lausanne). 2022;9:780000.

[25] Zhou C, Li Y, Wang Y, Fan Q, Dai L. Comparison of visual quality after SMILE correction of low-to-moderate myopia in different optical zones. Int Ophthalmol. 2023;43(10):3623-32.

[26] Luo W, Aruma A, Li M, Wang J, Xie J, Xiao X, Shen Y, Niu L, Wang X, Zhou X. Four-year visual outcomes and optical quality of SMILE and implantable collamer lens V4c (EVO-ICL) implantation for high myopia: a retrospective study. BMC Ophthalmol. 2023;23(1):341.
